# National Trends in Sadness, Suicidality, and COVID-19 Pandemic–Related Risk Factors Among South Korean Adolescents From 2005 to 2021

**DOI:** 10.1001/jamanetworkopen.2023.14838

**Published:** 2023-05-24

**Authors:** Ho Geol Woo, Sangil Park, Hyunju Yon, Seung Won Lee, Ai Koyanagi, Louis Jacob, Lee Smith, Wonyoung Cho, Chanyang Min, Jinseok Lee, Hayeon Lee, Rosie Kwon, Guillaume Fond, Laurent Boyer, Yoonie Yoonjung Joo, Yong Sung Choi, Seung-Geun Yeo, Sang Youl Rhee, Jae Il Shin, Dong Keon Yon

**Affiliations:** 1 Department of Neurology, Kyung Hee University Medical Center, Kyung Hee University College of Medicine, Seoul, South Korea; 2 Department of Medicine, Kyung Hee University College of Medicine, Seoul, South Korea; 3 Department of Precision Medicine, Sungkyunkwan University School of Medicine, Suwon, South Korea; 4 Research and Development Unit, Parc Sanitari Sant Joan de Deu, Network Centre for Biomedical Research in Mental Health, Health Institute Carlos III, Barcelona, Spain; 5 Catalan Institution for Research and Advanced Studies (ICREA), Pg. Lluis Companys, Barcelona, Spain; 6 Faculty of Medicine, University of Versailles Saint-Quentin-en-Yvelines, Montigny-le-Bretonneux, France; 7 Centre for Health, Performance and Wellbeing, Anglia Ruskin University, Cambridge, United Kingdom; 8 Center for Digital Health, Medical Science Research Institute, Kyung Hee University College of Medicine, Seoul, South Korea; 9 Department of Biomedical Engineering, Kyung Hee University, Yongin, South Korea; 10 Department of Biomedical Engineering, University of Michigan, Ann Arbor; 11 Assistance Publique-Hôpitaux de Marseille, Aix-Marseille University, CEReSS–Health Service Research and Quality of Life Center, Marseille, France; 12 Depression and Schizophrenia Expert Center, FondaMental Foundation, Creteil, France; 13 Institute of Data Science, Korea University, Seoul, South Korea; 14 Department of Pediatrics, Kyung Hee University Medical Center, Kyung Hee University College of Medicine, Seoul, South Korea; 15 Department of Otolaryngology–Head & Neck Surgery, Kyung Hee University Medical Center, Kyung Hee University College of Medicine, Seoul, South Korea; 16 Department of Endocrinology and Metabolism, Kyung Hee University School of Medicine, Seoul, South Korea; 17 Department of Pediatrics, Yonsei University College of Medicine, Seoul, South Korea

## Abstract

**Importance:**

Despite the COVID-19 pandemic’s effect on daily life, limited research exists on the prevalence and risk factors of suicidality and sadness among South Korean adolescents.

**Objectives:**

To examine whether the observed sadness and suicidality in the early to middle periods of the COVID-19 pandemic differed from the expected level and to investigate changes in risk factors for sadness and suicidality.

**Design, Setting, and Participants:**

This nationwide serial cross-sectional survey study used data on 1 109 776 Korean adolescents aged 13 to 18 years from the Korea Youth Risk Behavior Web-based Survey from 2005 to 2021.

**Exposure:**

The COVID-19 pandemic.

**Main Outcomes and Measures:**

The pattern of changes in the percentage or proportion of sadness or suicidality, as well as the risk factors for sadness or suicidality. The transitional effect of the COVID-19 pandemic was assessed using weighted odds ratios (wORs) or weighted beta coefficients with 95% CIs.

**Results:**

Between 2005 and 2021, 1 109 776 adolescents (mean [SD] age, 15.0 [1.7] years; 51.5% male adolescents; and 51.7% in grades 7-9 and 48.3% in grades 10-12) were included in the Korea Youth Risk Behavior Web-based Survey. The slope of the long-term trends in sadness and suicidality decreased in the prepandemic period (sadness: from 37.8% [95% CI, 37.4%-38.2%] in 2005-2007 to 26.1% [95% CI, 25.9%-26.4%] in 2016-2019; suicidality: from 23.0% [95% CI, 22.7%-23.3%] in 2005-2007 to 12.3% [95% CI, 12.1%-12.5%] in 2016-2019), whereas the slope increased during the COVID-19 pandemic (sadness: from 25.0% [95% CI, 24.5%-25.6%] in 2020 to 26.6% [95% CI, 26.1%-27.1%] in 2021; trend difference in β, 0.249 [95% CI, 0.236-0.262]; suicidality: from 10.7% [95% CI, 10.3%-11.1%] in 2020 to 12.5% [95% CI, 12.1%-12.9%] in 2021; trend difference in β, 0.328 [95% CI, 0.312-0.344]). The trends presented a similar tendency in the subgroups according to sex, school grade, residential area, smoking status, and current alcohol use. Compared with the prepandemic period, the risk factors associated with sadness during the pandemic were younger age (wOR, 0.907; 95% CI, 0.881-0.933), female sex (wOR, 1.031; 95% CI, 1.001-1.062), urban residence (wOR, 1.120; 95% CI, 1.087-1.153), current smoking status (wOR, 1.134; 95% CI, 1.059-1.216), and current alcohol use (wOR, 1.051; 95% CI, 1.002-1.102). Female sex (wOR, 1.064; 95% CI, 1.021-1.109), urban residence (wOR, 1.117; 95% CI, 1.074-1.162), and low economic status (wOR, 1.286; 95% CI, 1.180-1.403) were the risk factors significantly associated with suicidality after the COVID-19 pandemic began.

**Conclusions and Relevance:**

In this nationwide serial cross-sectional survey study of South Korean adolescents, the slope of the prevalence of sadness and suicidality increased during the COVID-19 pandemic after a decrease prior to the pandemic. The findings suggest that public health measures are needed to recognize vulnerable groups with risk factors and to prevent an increase in sadness and suicidality among adolescents during the COVID-19 pandemic.

## Key Points

**Question:**

How has the long-term trend of suicidality among Korean youths changed during the COVID-19 pandemic?

**Findings:**

In this nationwide serial cross-sectional survey study from 2005 to 2021 of 1 109 776 Korean adolescents aged 13 to 18 years, the slope of the long-term trends in suicidality decreased in the prepandemic period (from 23.0% in 2005-2007 to 12.3% in 2016-2019), whereas the slope increased during the COVID-19 pandemic (from 10.7% in 2020 to 12.5% in 2021). The risk factors for suicidality during the pandemic were younger age, female sex, urban residence, smoking, current alcohol use, and low economic status.

**Meaning:**

These results can help improve the understanding of suicidality during the COVID-19 pandemic.

## Teaser

This survey study of more than 1 million Korean adolescents examines whether the observed sadness and suicidality in the early to middle periods of the COVID-19 pandemic differed from the expected level and investigates changes in risk factors for sadness and suicidality.

## Introduction

The COVID-19 pandemic may exacerbate current mental health problems and lead to an increase in the incidence of mental disorders among children and adolescents due to the rare convergence of public health crisis, social isolation, and economic downturn.^1^ In the US, the growing trend of youth suicide rates and depression has become a major concern. According to the US Centers for Disease Control and Prevention, the suicide rate for young people aged 10 to 24 years increased by 57.4% from 2007 to 2018.^2^ Furthermore, previous studies have suggested that the spread of SARS-CoV-2 was associated with an increase in depression, anxiety, and suicidal ideation.^1,3^ However, other conflicting reports suggested that suicidality decreased or remained stable during the COVID-19 pandemic.^4,5,6,7^ Furthermore, during the early months of the COVID-19 pandemic, overall suicide rates in high-income and upper-middle–income countries were largely constant or declining.^7^ Rapid financial support was provided during the early stages of the pandemic to mitigate the economic consequences of the pandemic and prevent increased suicide rates. However, this may change as government support decreases over time and the economy worsens.

According to available data, Korean adolescents have experienced high rates of mental health problems, including suicidal ideation, suicide attempts, and self-injury,^8^ due to a number of factors, including academic pressure, social isolation, and cultural stigma surrounding mental health.^9^ In addition, there have been concerns about the increase in overdose deaths among Korean young people.^10^ Addressing the underlying causes of these issues, providing access to mental health resources, and reducing the associated stigma are important steps toward improving the mental health and well-being of Korean adolescents.

Nevertheless, to our knowledge, there are few systemic data indicating a connection between COVID-19 and an increased risk of suicidality.^1,3^ Thus, this study aimed to analyze trends using nationally representative survey data from 2005 to 2021 involving South Korean adolescents. We evaluated the association of the pandemic with sadness and suicidality by comparing the trends and risk factors of sadness and suicidality during the prepandemic period (2005-2019) and pandemic period (2020-2021). In addition, this study investigated whether the levels of sadness and suicidality observed during the pandemic differed from the expected levels.

## Methods

### Sample Selection and Data Collection

This study was designed and conducted in accordance with the American Association for Public Opinion Research (AAPOR) reporting guideline for investigative research. Our methods adhere to these guidelines by providing information on the study design, sampling procedures, response rates, and measures used in this research.^11^ The Kyung Hee University and the Korea Disease Control and Prevention Agency approved the study protocol. Written informed consent was obtained from all participants at enrollment.

We used the nationally representative data of 1 109 776 adolescents from the Korea Youth Risk Behavior Web-based Survey (KYRBS), which was conducted annually between 2005 and 2021 and overseen by the Ministry of Education and the Korea Disease Control and Prevention Agency. This survey is an ongoing national cross-sectional survey to assess health risk behaviors among middle- and high-school students. The sampling strategy has been designed each year. It is possible that some of the adolescents had responded to the same survey more than once. First, the population was stratified using region and school level as stratification variables. Second, proportional assignment was conducted to match the ratio of population to sample composition. Third, for sampling, the first extraction unit was school and the second extraction unit was class using stratified cluster sampling (eFigure 1 in Supplement 1).^12^ Then, the level of youth health behavior was estimated by weighting the students who participated in the survey to represent Korean youth. Through this method, approximately 2% of students conducted the survey as representatives.

As a result, 30 015 middle school respondents and 24 833 high school respondents represent 1 339 993 middle school students and 1 289 595 high school students nationwide in 2021. Adolescents in middle and high schools between 13 and 18 years of age were recruited and voluntarily participated in the web-based survey at their individual schools (mean response rate, 95%).^13^

### Covariate Definitions

The variables included age, grade (7-9 [middle school] and 10-12 [high school]), sex, body mass index (calculated as weight in kilograms divided by height in meters squared; continuous value), residential areas (rural areas [Chungbuk, Chungnam, Gangwon, Gyeongbuk, Gyeongnam, Gyeonggi, Jeonbuk, Jeonnamand, and Jeju] and urban areas [Seoul, Busan, Daegu, Incheon, Gwangju, Daejeon, Ulsan, and Sejong]), current smoking status, and parents’ highest educational level (high school or lower, college or higher, and unknown). Furthermore, we defined current drinkers as those who experienced alcohol drinking on 1 to 30 days within 1 month. In the survey, the adolescent participants were asked to respond to how many days they consumed an alcohol drink within the last 30 days: none, 1 to 2 days, 3 to 5 days, 6 to 9 days, 10 to 19 days, 20 to 29 days, and every day. We sorted these responses into 2 categories: none and current drinker (alcohol drinking on 1-30 days). Socioeconomic status was classified subjectively into high, middle-high, middle, middle-low, and low using the single question, “What do you perceive as your household economic status?”^14^ Furthermore, academic performance was measured by the question, “During the past year, how was your school achievement?” The response was classified into 5 categories: (1) high, (2) middle-high, (3) middle, (4) middle-low, and (5) low on average within the last 12 months.^15^

### End Points

The main objective was to ascertain whether the COVID-19 pandemic had any association with suicidality and sadness trends across 17 years. We classified the pandemic periods as 2 categories: the years 2020 and 2021. The term *suicidality* was defined as suicidal thoughts, plans, and attempted suicide within 12 months for questionnaires as in previous studies.^15,16,17^ The same question assessed suicidal thoughts over the entire time period: “Have you ever thought seriously about trying to kill yourself in the past 12 months?” For respondents who reported having suicidal thoughts in the past 12 months, 2 additional questions evaluated suicide plans and attempts: “Did you make any plans to kill yourself in the past 12 months?” and “Did you try to kill yourself in the last 12 months?” Sadness was defined as feelings of despair at least once within the last 12 months.^17^ One question assessed past 12-month sadness: “In the past 12 months, have you felt so much sadness that you stopped your daily life for 2 weeks?”

In addition, we compared the trend shift for sadness and suicidality in each subgroup (by sex, grade, location of residence, and smoking status) before and during the COVID-19 pandemic and used weighted means calculated through inverse probability weighting (eFigure 1 in Supplement 1).

### Statistical Analysis

To assess the pattern of changes in the percentage or proportion of sadness and suicidal ideation, we used data from the KYRBS between 2005 and 2021, stratified by sex, grade, residence area, and smoking status, and weighted mean values with 95% CIs and crude numbers with percentages. Time periods were divided into 3- to 4-year cycles for the prepandemic period (2005-2007, 2008-2011, 2012-2015, and 2016-2019) and the COVID-19 pandemic period (years 2020 and 2021) to stabilize the longitudinal trend.^18^

This repeated cross-sectional survey study confirmed the statistical significance of changes and trends over time through graphical methods^19^ and regression analysis.^20^ Using binary logistic regression models, we conducted weighted complex sampling analysis to represent the nationwide population. The results of these analyses are shown as weighted odds ratios (wORs) for prevalence or weighted β coefficients with 95% CIs (eFigure 1 in Supplement 1).^21^ The KYRBS cycle was our preferred option for binary regression (latest prepandemic period [2016-2019] vs pandemic period [2020-2021]).

Using the Fisher exact test for categorical variables and the *t* test for continuous variables, it was possible to compare the changes in risk factors between the pre–COVID-19 pandemic (2005-2019) and COVID-19 pandemic (2020-2021) periods.^22^ In addition, we confirmed the linearity assumption of body mass index using the Box-Tidwell test.^23^ If the tolerance is less than 1, the variance inflation factor is 10 or higher, or the correlation coefficient is 0.9 or higher through linear regression analysis, then the variable is excluded as a multicollinearity variable. To evaluate independent risk factors for sadness and suicidality, variables with *P* < .10 from the univariate logistic regression analysis were used to input variables. Weighted ORs with 95% CIs were provided to determine whether the magnitude of the risk factors differed before and after the pandemic.

All analyses were performed using SPSS, version 25.0 (IBM Corp) and R software, version 4.2.1 (R Group for Statistical Computing). A 2-sided *P* < .05 was considered statistically significant.

## Results

Crude data on 1 109 776 adolescents (mean [SD] age, 15.0 [1.7] years; 51.7% in grades 7-9 and 48.3% in grades 10-12; and 106 979 adolescents during the pandemic) were collected in the KYRBS from 2005 to 2021, including 572 055 males (crude, 51.5%; weighted, 52.4% [95% CI, 51.8%-53.1%]). Table 1 and eTable 1 in Supplement 1 provide an overview of the demographic characteristics. The weighted estimated mean age was 15.04 years (95% CI, 15.03-15.05 years), 50.3% (95% CI, 49.9%-50.6%) were in grades 7 to 9 (middle school), and 49.7% (95% CI, 49.4%-50.1%) were in grades 10 to 12 (high school).

**Table 1.** Demographic Characteristics of Participating Adolescents in the Korea Youth Risk Behavior Web-based Survey, 2005-2021

Variable	Weighted sample					
	Overall	2005-2007	2008-2011	2012-2015	2016-2019	COVID-19 pandemic (2020-2021)
Estimated weighted sample size, mean (SD)	56 122 639 (226 267)	10 518 980 (109 115)	15 148 697 (126 299)	13 901 009 (89 830)	11 418 859 (89 830)	5 135 095 (58 931)
Proportion (95% CI)	100.0 (100.0-100.0)	18.7 (18.4-19.1)	27.0 (26.6-27.4)	24.8 (24.4-25.1)	20.3 (20.1-20.6)	9.1 (9.0-9.4)
Age, mean (95% CI), y	15.04 (15.03-15.05)	14.88 (14.85-14.91)	15.08 (15.06-15.11)	14.99 (14.97-15.01)	15.12 (15.09-15.14)	15.20 (15.17-15.24)
School grade, proportion (95% CI)						
7-9 (Middle school)	50.3 (49.9-50.6)	56.3 (55.3-57.2)	50.6 (49.8-51.4)	48.4 (47.7-49.2)	46.4 (45.6-47.2)	50.4 (49.3-51.6)
10-12 (High school)	49.7 (49.4-50.1)	43.7 (42.8-44.7)	49.4 (48.6-50.2)	51.6 (50.8-52.3)	53.6 (52.8-54.4)	49.6 (48.4-50.7)
Sex, proportion (95% CI)						
Male	52.4 (51.8-53.1)	52.9 (51.2-54.6)	52.8 (51.4-54.2)	52.3 (50.9-53.7)	52.1 (50.7-53.4)	51.9 (50.2-53.6)
Female	47.6 (46.9-48.2)	47.1 (45.4-48.8)	47.7 (45.8-48.6)	47.7 (46.3-49.1)	47.9 (46.6-49.3)	48.1 (46.4-49.8)
BMI, mean (95% CI)	20.79 (20.78-20.81)	20.48 (20.45-20.51)	20.48 (20.45-20.50)	20.73 (20.70-20.75)	21.25 (21.22-21.27)	21.55 (21.51-21.60)
Residence, proportion (95% CI)						
Rural	45.9 (45.5-46.3)	47.3 (46.3-48.3)	50.1 (49.3-51.0)	44.0 (43.3-44.7)	43.0 (42.3-43.8)	42.2 (41.1-43.2)
Urban	54.1 (53.7-54.5)	52.7 (51.7-53.7)	49.9 (49.0-50.7)	56.0 (55.3-56.7)	57.0 (56.2-57.7)	57.8 (56.8-58.9)
Smoking, proportion (95% CI)	9.5 (9.4-9.7)	12.3 (11.9-12.6)	12.2 (11.9-12.5)	9.3 (9.0-9.6)	6.2 (6.0-6.4)	4.3 (4.1-4.5)
Current alcohol use, proportion (95% CI)	19.3 (19.2-19.5)	27.6 (27.2-28.1)	21.6 (12.2-21.9)	17.0 (16.7-17.3)	15.4 (15.1-15.7)	10.5 (10.2-10.8)
Sadness, proportion (95% CI)	31.5 (31.3-31.6)	37.8 (37.4-38.2)	36.4 (36.1-36.7)	27.8 (27.5-28.1)	26.1 (25.9-26.4)	25.8 (25.4-26.2)
Suicidality, proportion (95% CI)	16.6 (16.5-16.7)	23.0 (22.7-23.3)	18.9 (18.7-19.2)	14.7 (14.5-14.9)	12.3 (12.1-12.5)	11.6 (11.3-11.9)
Suicide attempt, proportion	3.6 (3.6-3.7)	5.1 (5.0-5.3)	4.4 (4.3-4.5)	3.2 (3.1-3.3)	2.5 (2.4-2.6)	2.0 (1.9-2.1)
Highest educational level of parents, proportion (95% CI)						
High school or lower	46.4 (46.1-46.7)	62.7 (62.0-63.4)	55.6 (55.1-56.2)	45.4 (45.0-45.9)	31.7 (31.2-32.1)	21.1 (20.6-21.7)
College or higher	38.5 (38.2-38.8)	27.2 (26.5-28.0)	33.9 (33.3-34.5)	42.1 (41.5-42.6)	47.8 (47.2-48.4)	44.9 (44.2-45.6)
Unknown	15.1 (15.0-15.2)	10.1 (9.8-10.3)	10.4 (10.2-10.7)	12.5 (12.3-12.7)	20.5 (20.2-20.9)	34.0 (33.4-34.5)
Economic level, proportion (95% CI)						
High	8.1 (8.0-8.2)	7.3 (7.1-7.5)	6.3 (6.1-6.5)	7.6 (7.4-7.8)	10.4 (10.2-10.7)	11.0 (10.7-11.3)
Middle-high	27.0 (26.8-27.2)	31.2 (30.7-31.6)	23.2 (22.9-23.5)	25.6 (25.3-25.9)	29.0 (28.7-29.3)	29.2 (28.7-29.6)
Middle	46.7 (46.6-46.9)	43.9 (43.5-44.3)	47.2 (47.0-47.5)	47.6 (47.3-47.9)	46.9 (46.6-47.3)	48.3 (47.8-48.8)
Middle-low	14.3 (14.2-14.5)	14.0 (13.7-14.3)	17.7 (17.5-18.0)	15.3 (15.1-15.5)	11.2 (11.1-11.4)	9.6 (9.3-9.8)
Low	3.8 (3.8-3.9)	3.7 (3.6-3.8)	5.6 (5.5-5.7)	3.9 (3.9-4.1)	2.3 (2.3-2.4)	1.9 (1.8-2.0)
School performance, proportion (95% CI)						
High	12.2 (12.1-12.3)	13.5 (13.3-13.8)	11.3 (11.1-11.5)	11.5 (11.3-11.7)	13.1 (12.9-13.3)	12.4 (12.1-12.7)
Middle-high	25.5 (25.4-25.6)	29.1 (28.8-29.3)	24.0 (23.8-24.2)	24.6 (24.4-24.7)	25.5 (25.3-25.7)	24.8 (24.4-25.1)
Middle	28.5 (28.4-28.6)	29.3 (29.0-29.6)	27.2 (27.0-27.5)	27.9 (27.7-28.1)	29.4 (29.2-29.6)	30.7 (30.4-31.0)
Middle-low	23.4 (23.3-23.5)	20.3 (20.1-20.6)	25.6 (25.4-25.8)	24.5 (24.3-24.7)	22.3 (22.1-22.6)	22.4 (22.1-22.7)
Low	10.4 (10.3-10.5)	7.8 (7.6-8.0)	11.9 (11.7-12.0)	11.6 (11.4-11.7)	9.7 (9.5-9.8)	9.7 (9.5-9.9)

Abbreviation: BMI, body mass index (calculated as weight in kilograms divided by height in meters squared).

The Figure and eFigure 2 in Supplement 1 show the trends in the prevalence of sadness and suicidality among Korean adolescents from 2005 to 2021. The prevalence rates for both items decreased steadily over time, with different slopes except during the COVID-19 pandemic. Table 2 and Table 3 present the trends and proportions of sadness and suicidality from 2005 to 2021 with regression slope coefficients denoted by β. The slope of the long-term trends in sadness and suicidality decreased in the prepandemic period (sadness: from 37.8% [95% CI, 37.4%-38.2%] in 2005-2007 to 26.1% [95% CI, 25.9%-26.4%] in 2016-2019 [Table 2]; suicidality: from 23.0% [95% CI, 22.7%-23.3%] in 2005-2007 to 12.3% [95% CI, 12.1%-12.5%] in 2016-2019 [Table 3]), whereas the slope increased during the COVID-19 pandemic (sadness: from 25.0% [95% CI, 24.5%-25.6%] in 2020 to 26.6% [95% CI, 26.1%-27.1%] in 2021; trend difference in β, 0.249 [95% CI, 0.236-0.262] [Table 2]; suicidality: from 10.7% [95% CI, 10.3%-11.1%] in 2020 to 12.5% [95% CI, 12.1%-12.9%] in 2021; trend difference in β, 0.328 [95% CI, 0.312-0.344] [Table 3]). The slope of the 17-year trends in the overall prevalence of sadness and suicidality presented a similar tendency in subgroups according to sex, grade, residential area, smoking, and current alcohol consumption (Table 2 and Table 3).

**Figure.**
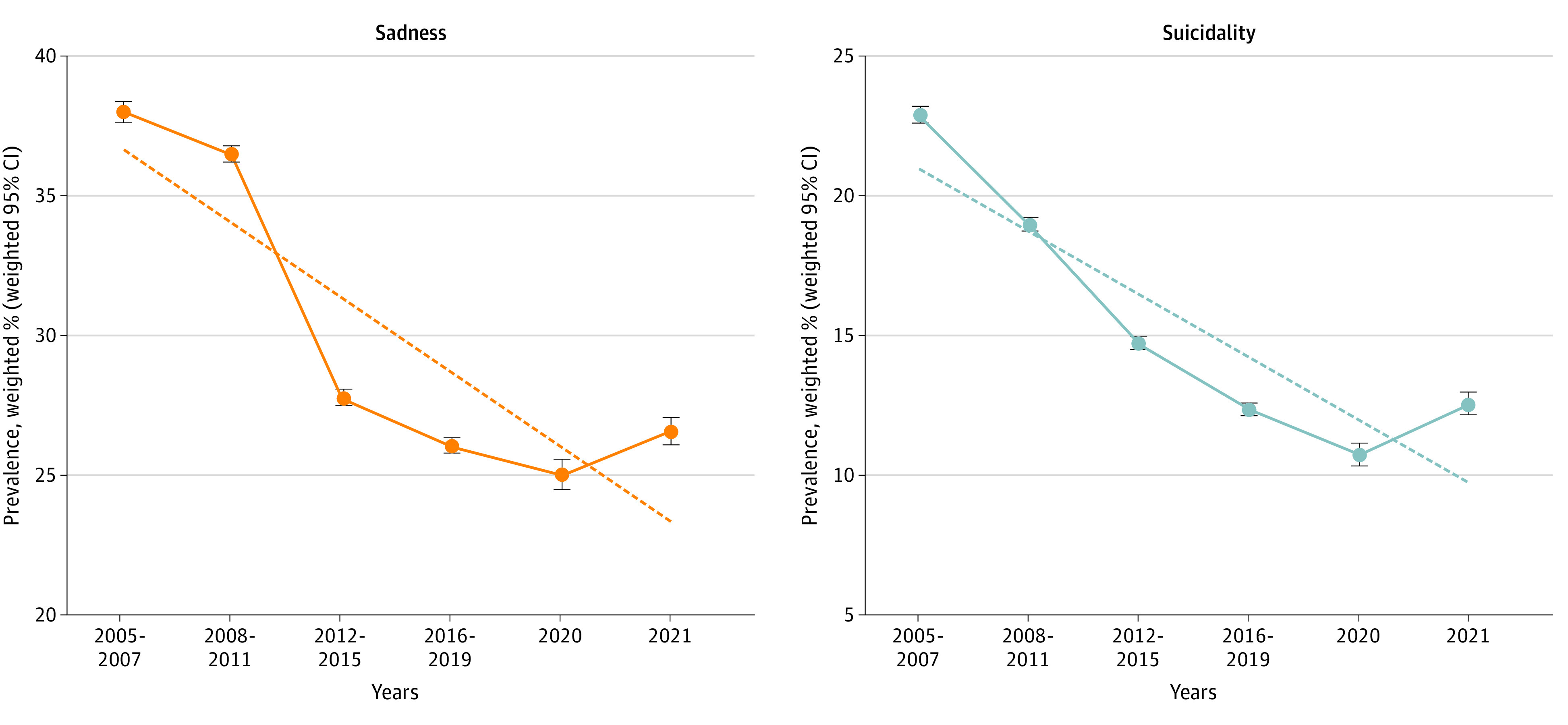
Nationwide 17-Year Trends and Prevalence of Sadness and Suicidality Among 1 Million Korean Adolescents, 2005-2021 The trend line represented by a dotted line includes the period from 2015 to 2021. Error bars indicate weighted 95% CIs.

**Table 2.** National Weighted Prevalence and Trends for Sadness Among the Adolescent Population in South Korea, 2005-2021

Variable	Trends in sadness, weighted % (95% CI)							Trend analysis					2016-2019 [Reference] vs 2020-2021	
	2005-2007	2008-2011	2012-2015	2016-2019	2020-2021	2020	2021	Trend before pandemic, β (95% CI)^a^	*P* value	Trend after pandemic, β (95% CI)^a^	*P* value	Trend difference in β (95% CI)	wOR (95% CI)^b^	*P* value
Overall	37.8 (37.4 to 38.2)	36.4 (36.1 to 36.7)	27.8 (27.5 to 28.1)	26.1 (25.9 to 26.4)	25.8 (25.4 to 26.2)	25.0 (24.5 to 25.6)	26.6 (26.1 to 27.1)	−0.229 (−0.237 to −0.222)	<.001	0.020 (0.010 to 0.031)	<.001	0.249 (0.236 to 0.262)	0.989 (0.967 to 1.010)	.30
Sex														
Male	33.3 (32.9 to 33.8)	31.4 (31.1 to 31.7)	23.0 (22.7 to 23.3)	20.8 (20.5 to 21.1)	21.1 (20.7 to 21.6)	19.9 (19.3 to 20.5)	22.3 (21.7 to 22.9)	−0.265 (−0.274 to −0.225)	<.001	0.036 (0.023 to 0.049)	<.001	0.301 (0.273 to 0.329)	1.014 (0.989 to 1.041)	.30
Female	42.8 (42.3 to 43.4)	41.9 (41.6 to 42.3)	33.0 (32.7 to 33.4)	31.9 (31.6 to 32.3)	30.9 (30.4 to 31.4)	30.6 (29.8 to 31.3)	31.2 (30.5 to 31.9)	−0.205 (−0.214 to −0.196)	<.001	0.007 (−0.004 to 0.019)	.22	0.212 (0.197 to 0.227	0.969 (0.950 to 0.988)	.001
School grade														
7-9 (Middle school)	34.6 (34.1 to 35.1)	33.3 (32.9 to 33.7)	25.7 (25.3 to 26.0)	24.3 (23.9 to 24.7)	24.4 (23.9 to 24.9)	22.8 (22.2 to 23.5)	25.8 (25.1 to 26.5)	−0.213 (−0.223 to −0.203)	<.001	0.040 (0.027 to 0.054)	<.001	0.253 (0.236 to 0.270)	1.004 (0.978 to 1.031)	.76
10-12 (High school)	41.9 (41.4 to 42.4)	39.6 (39.1 to 40.0)	29.8 (29.4 to 30.2)	27.7 (27.3 to 28.1)	27.3 (26.7 to 27.9)	27.2 (26.3 to 28.1)	27.4 (26.6 to 28.2)	−0.260 (−0.270 to −0.250)	<.001	0.003 (−0.012 to 0.018)	.70	0.263 (0.245 to 0.281)	0.986 (0.955 to 1.017)	.36
Residence														
Rural	37.9 (37.4 to 38.4)	36.8 (36.4 to 37.2)	27.9 (27.5 to 28.3)	25.8 (25.4 to 26.2)	24.9 (24.4 to 25.5)	24.3 (23.4 to 25.1)	25.6 (24.8 to 26.4)	−0.232 (−0.242 to −0.222)	<.001	0.018 (0.002 to 0.034)	.03	0.250 (0.231 to 0.269)	0.965 (0.939 to 0.991)	.001
Urban	37.8 (37.2 to 38.3)	36.0 (35.5 to 36.4)	27.7 (27.4 to 28.1)	26.4 (26.0 to 26.8)	26.5 (25.9 to 27.0)	25.6 (24.9 to 26.3)	27.3 (26.6 to 28.0)	−0.226 (−0.236 to −0.215)	<.001	0.022 (0.009 to 0.035)	.001	0.248 (0.231 to 0.265)	1.004 (0.978 to 1.030)	.77
Smoking														
No	35.9 (35.5 to 36.3)	34.6 (34.3 to 34.9)	26.4 (26.1 to 26.7)	25.2 (25.0 to 25.5)	25.0 (24.6 to 25.4)	24.2 (23.6 to 24.8)	25.8 (25.2 to 26.3)	−0.219 (−0.227 to −0.211)	<.001	0.021 (0.011 to 0.031)	<.001	0.240 (0.227 to 0.253)	0.992 (0.974 to 1.011)	.41
Yes	51.6 (50.7 to 52.4)	49.0 (48.3 to 49.8)	41.5 (40.8 to 42.3)	39.8 (38.8 to 40.8)	44.3 (42.6 to 46.1)	43.6 (41.2 to 46.1)	44.9 (42.5 to 47.4)	−0.176 (−0.192 to −0.160)	<.001	0.013 (−0.022 to 0.048)	.47	0.189 (0.151 to 0.227)	1.113 (1.106 to 1.166)	<.001
Current alcohol use														
No	33.8 (33.4 to 34.2)	33.3 (33.0 to 33.6)	25.4 (25.2 to 25.7)	24.0 (23.7 to 24.3)	24.2 (23.8 to 24.6)	23.4 (22.8 to 23.9)	25.0 (24.4 to 25.5)	−0.206 (−0.214 to −0.199)	<.001	0.022 (0.011 to 0.032)	<.001	0.228 (0.021 to 0.435)	1.008 (0.988 to 1.029)	.43
Yes	48.4 (47.8 to 49.0)	47.6 (47.1 to 48.2)	39.3 (38.8 to 39.9)	37.8 (37.2 to 38.4)	39.8 (38.7 to 40.9)	39.2 (37.6 to 40.7)	40.4 (38.8 to 42.0)	−0.182 (−0.195 to −0.170)	<.001	0.013 (−0.010 to 0.036)	.27	0.195 (0.169 to 0.221)	1.053 (1.020 to 1.087)	.002

Abbreviation: wOR, weighted odds ratio.

^a^ Calculated using linear regression.

^b^ Derived using logistic regression; this model included the Korea Youth Risk Behavior Web-based Survey cycle (2016-2019 [reference] vs 2020-2021 [COVID-19 pandemic]) as a categorical variable.

**Table 3.** National Weighted Prevalence and Trends of Suicidality Among the Adolescent Population in South Korea, 2005-2021

Variable	Trends in suicidality, weighted % (95% CI)							Trend analysis					2016-2019 [Reference] vs 2020-2021	
	2005-2007	2008-2011	2012-2015	2016-2019	2020-2021	2020	2021	Trend before pandemic, β (95% CI)^a^	*P* value	Trend after pandemic, β (95% CI)^a^	*P* value	Trend difference in β (95% CI)	wOR (95% CI)^b^	*P* value
Overall	23.0 (22.7 to 23.3)	18.9 (18.7 to 19.2)	14.7 (14.5 to 14.9)	12.3 (12.1 to 12.5)	11.6 (11.3 to 11.9)	10.7 (10.3 to 11.1)	12.5 (12.1 to 12.9)	−0.284 (−0.293 to −0.276)	<.001	0.044 (0.031 to 0.057)	<.001	0.328 (0.312 to 0.344)	0.943 (0.915 to 0.972)	<.001
Sex														
Male	18.5 (18.2 to 18.8)	15.1 (14.8 to 15.3)	11.7 (11.5 to 11.9)	9.1 (8.9 to 9.3)	8.7 (8.4 to 9.0)	7.9 (7.5 to 8.4)	9.4 (9.0 to 9.8)	−0.299 (−0.310 to −0.287)	<.001	0.046 (0.028 to 0.065)	<.001	0.345 (0.323 to 0.367)	0.956 (0.918 to 0.996)	.03
Female	28.0 (27.6 to 28.4)	23.3 (22.9 to 23.6)	18.0 (17.7 to 18.2)	15.7 (15.5 to 16.0)	14.8 (14.4 to 15.2)	13.7 (13.1 to 14.2)	15.8 (15.3 to 16.4)	−0.281 (−0.292 to −0.271)	<.001	0.043 (0.028 to 0.059)	<.001	0.324 (0.305 to 0.343)	0.943 (0.914 to 0.973)	<.001
School grade														
7-9 (Middle school)	22.9 (22.4 to 23.3)	19.2 (18.8 to 19.5)	15.1 (14.9 to 15.4)	12.9 (12.6 to 13.1)	11.7 (11.6 to 12.0)	10.1 (9.6 to 10.6)	13.2 (12.7 to 13.8)	−0.265 (−0.276 to −0.253)	<.001	0.076 (0.059 to 0.093)	<.001	0.341 (0.320 to 0.362)	0.907 (0.884 to 0.931)	<.001
10-12 (High school)	23.1 (22.7 to 23.6)	18.7 (18.4 to 19.1)	14.2 (14.0 to 14.5)	11.8 (11.6 to 12.1)	11.5 (11.1 to 11.9)	11.3 (10.7 to 11.9)	11.8 (11.2 to 12.3)	−0.300 (−0.312 to −0.287)	<.001	0.012 (−0.008 to 0.031)	.25		0.975 (0.936 to 1.015)	.21
Residence														
Rural	23.0 (22.5 to 23.4)	19.0 (18.7 to 19.3)	14.9 (14.7 to 15.2)	12.3 (12.0 to 12.6)	11.1 (10.7 to 11.5)	10.3 (9.7 to 10.9)	11.9 (11.3 to 12.5)	−0.274 (−0.286 to −0.262)	<.001	0.040 (0.019 to 0.061)	<.001	0.314 (0.290 to 0.338)	0.902 (0.864 to 0.943)	<.001
Urban	23.0 (22.5 to 23.5)	18.9 (18.5 to 19.2)	14.5 (14.2 to 14.7)	12.3 (12.1 to 12.6)	12.0 (11.6 to 12.3)	11.0 (10.5 to 11.5)	13.0 (12.5 to 13.5)	−0.292 (−0.304 to −0.279)	<.001	0.047 (0.030 to 0.063)	<.001	0.339 (0.318 to 0.360)	0.976 (0.941 to 1.011)	.17
Smoking														
No	21.6 (21.2 to 21.9)	17.7 (17.4 to 17.9)	13.7 (13.6 to 13.9)	11.8 (11.6 to 12.0)	11.1 (10.8 to 11.4)	10.2 (9.9 to 10.6)	12.0 (11.6 to 12.4)	−0.275 (−0.285 to −0.266)	<.001	0.045 (0.031 to 0.058)	<.001	0.320 (0.303 to 0.337)	0.941 (0.911 to 0.971)	<.001
Yes	33.1 (32.3 to 33.9)	28.0 (27.3 to 28.6)	23.8 (23.1 to 24.4)	19.9 (19.1 to 20.7)	22.4 (21.0 to 23.9)	21.0 (19.2 to 22.9)	23.8 (21.7 to 26.0)	−0.220 (−0.238 to −0.202)	<.001	0.041 (0.066 to 0.082)	.04	0.261 (0.241 to 0.281)	1.126 (1.043 to 1.215)	.002
Current alcohol use														
No	20.5 (20.2 to 20.8)	17.0 (16.8 to 17.2)	13.2 (13.0 to 13.4)	11.2 (11.0 to 11.4)	10.7 (10.4 to 10.9)	9.7 (9.3 to 10.1)	11.6 (11.2 to 12.0)	−0.267 (−0.277 to −0.258)	<.001	0.051 (0.037 to 0.064)	<.001	0.318 (0.301 to 0.335)	0.955 (0.928 to 0.984)	.002
Yes	29.4 (28.9 to 30.0)	26.0 (25.5 to 26.6)	21.8 (21.3 to 22.3)	18.2 (17.7 to 18.7)	19.6 (18.8 to 20.5)	19.2 (18.0 to 20.5)	20.0 (18.8 to 21.3)	−0.227 (−0.241 to −0.212)	<.001	0.013 (−0.016 to 0.041)	.38	0.240 (0.208 to 0.272)	1.077 (1.023 to 1.134)	.005

Abbreviation: wOR, weighted odds ratio.

^a^ Calculated using linear regression.

^b^ Derived using logistic regression; this model included the Korea Youth Risk Behavior Web-based Survey cycle (2016-2019 [reference] vs 2020-2021 [COVID-19 pandemic]) as a categorical variable.

Compared with the prepandemic period, after the COVID-19 pandemic began, younger age (reference, middle school grade: wOR, 0.907; 95% CI, 0.881-0.933; *P* < .001), female sex (reference, male: wOR, 1.031; 95% CI, 1.001-1.062; *P* = .04), urban residence (reference, rural residence: wOR, 1.120; 95% CI, 1.087-1.153; *P* < .001), current smoking status (reference, no smoking: wOR, 1.134; 95% CI, 1.059-1.216; *P* < .001), and current alcohol use (reference, no alcohol use: wOR, 1.051; 95% CI, 1.002-1.102; *P* = .04) were the risk factors significantly associated with sadness (Table 4). Female sex (reference, male: 1.064; 95% CI, 1.021-1.109; *P* = .003) and urban residence (reference, rural residence: wOR, 1.117; 95% CI, 1.074-1.162; *P* < .001) were the risk factors significantly associated with suicidality after the COVID-19 pandemic began. In addition, compared with the highest economic level, lower economic level was a risk factor associated with suicidality, with the largest pandemic-to-prepandemic ratio of wOR at the middle-low economic level (1.286; 95% CI, 1.180-1.403) (Table 4; eTables 2-5 in Supplement 1).

**Table 4.** Adjusted and Weighted Logistic Regression Analysis

Variable	Pre–COVID-19 pandemic (2005-2019) (estimated weighted No. [SD], 50 987 544 [218 458])		COVID-19 pandemic (2020-2021) (estimated weighted No. [SD], 5 135 095 [58 931])		Ratio of wOR (pandemic to prepandemic) (95% CI)	*P* value
	wOR (95% CI)^a^	*P* value	wOR (95% CI)^a^	*P* value		
**Sadness related** ^b^						
School grade						
7-9 (Middle school)	1 [Reference]	NA	1 [Reference]	NA	NA	NA
10-12 (High school)	1.270 (1.259-1.281)	<.001	1.152 (1.121-1.184)	<.001	0.907 (0.881-0.933)	<.001
Sex						
Male	1 [Reference]	NA	1 [Reference]	NA	NA	NA
Female	1.755 (1.743-1.772)	<.001	1.809 (1.756-1.860)	<.001	1.031 (1.001-1.062)	.04
BMI	0.998 (0.997-0.999)	<.001	1.000 (0.996-1.004)	.85	1.002 (0.998-1.006)	.34
Residence						
Rural	1 [Reference]	NA	1 [Reference]	NA	NA	NA
Urban	0.965 (0.957-0.974)	<.001	1.081 (1.050-1.112)	<.001	1.120 (1.087-1.153)	<.001
Current smoking	1.541 (1.518-1.565)	<.001	1.748 (1.634-1.870)	<.001	1.134 (1.059-1.216)	<.001
Current alcohol use	1.671 (1.651-1.690)	<.001	1.756 (1.677-1.838)	<.001	1.051 (1.002-1.102)	.04
Highest educational level of parents						
High school or lower	1.141 (1.131-1.152)	<.001	1.129 (1.090-1.170)	<.001	0.989 (0.954-1.026)	.57
College or higher	1 [Reference]	NA	1 [Reference]	NA	NA	NA
Unknown	0.863 (0.851-0.874)	<.001	0.965 (0.935-0.996)	.03	1.118 (1.080-1.157)	<.001
Economic level						
High	1 [Reference]	NA	1 [Reference]	NA	NA	NA
Middle-high	0.951 (0.934-0.969)	<.001	0.974 (0.925-1.026)	.32	1.024 (0.969-1.082)	.39
Middle	0.946 (0.929-0.963)	<.001	0.922 (0.878-0.969)	.001	0.975 (0.925-1.027)	.34
Middle-low	1.332 (1.305-1.359)	<.001	1.360 (1.279-1.447)	<.001	1.021 (0.957-1.090)	.53
Low	1.836 (1.788-1.885)	<.001	1.951 (1.767-2.154)	<.001	1.063 (0.959-1.177)	.25
School performance						
High	1 [Reference]	NA	1 [Reference]	NA	NA	NA
Middle-high	1.092 (1.075-1.109)	<.001	1.046 (0.994-1.101)	.09	0.958 (0.908-1.010)	.11
Middle	1.180 (1.162-1.199)	<.001	1.121 (1.066-1.179)	<.001	0.950 (0.901-1.001)	.06
Middle-low	1.374 (1.352-1.397)	<.001	1.352 (1.283-1.425)	<.001	0.984 (0.931-1.040)	.57
Low	1.598 (1.568-1.629)	<.001	1.689 (1.588-1.795)	<.001	1.057 (0.991-1.127)	.09
**Suicidality related** ^c^						
School grade						
7-9 (Middle school)	1 [Reference]	NA	1 [Reference]	NA	NA	NA
10-12 (High school)	0.960 (0.950-0.970)	<.001	0.981 (0.945-1.018)	.31	1.022 (0.983-1.062)	.27
Sex						
Male	1 [Reference]	NA	1 [Reference]	NA	NA	NA
Female	1.882 (1.861-1.903)	<.001	2.002 (1.924-2.083)	<.001	1.064 (1.021-1.109)	.003
BMI	1.001 (0.999-1.002)	.34	1.002 (0.997-1.007)	.40	1.001 (0.996-1.006)	.71
Residence						
Rural	1 [Reference]	NA	1 [Reference]	NA	NA	NA
Urban	0.954 (0.943-0.964)	<.001	1.066 (1.026-1.107)	.001	1.117 (1.074-1.162)	<.001
Current smoking	1.667 (1.638-1.697)	<.001	1.716 (1.579-1.863)	<.001	1.029 (0.946-1.120)	.50
Current alcohol use	1.687 (1.664-1.710)	<.001	1.759 (1.660-1.865)	<.001	1.043 (0.982-1.107)	.17
Highest educational level of parents						
High school or lower	1.134 (1.122-1.147)	<.001	1.093 (1.042-1.146)	<.001	0.964 (0.918-1.012)	.14
College or higher	1 [Reference]	NA	1 [Reference]	NA	NA	NA
Unknown	0.909 (0.893-0.924)	<.001	0.882 (0.844-0.921)	<.001	0.970 (0.924-1.018)	.22
Economic level						
High	1 [Reference]	NA	1 [Reference]	NA	NA	NA
Middle-high	0.961 (0.939-0.984)	.001	1.092 (1.014-1.175)	.02	1.136 (1.052-1.228)	.001
Middle	0.961 (0.940-0.983)	.001	1.076 (1.002-1.155)	.04	1.120 (1.039-1.206)	.003
Middle-low	1.484 (1.448-1.522)	<.001	1.909 (1.757-2.074)	<.001	1.286 (1.180-1.403)	<.001
Low	2.200 (2.213-2.269)	<.001	2.743 (2.433-3.092)	<.001	1.247 (1.105-1.406)	<.001
School performance						
High	1 [Reference]	NA	1 [Reference]	NA	NA	NA
Middle-high	1.015 (0.996-1.036)	.13	0.941 (0.878-1.009)	.94	0.927 (0.862-0.997)	.04
Middle	1.041 (1.021-1.062)	<.001	0.917 (0.856-0.982)	.01	0.881 (0.820-0.946)	.001
Middle-low	1.203 (1.179-1.227)	<.001	1.112 (1.036-1.193)	.003	0.924 (0.859-0.995)	.04
Low	1.386 (1.355-1.418)	<.001	1.402 (1.294-1.520)	<.001	1.012 (0.930-1.100)	.79

Abbreviations: BMI, body mass index (calculated as weight in kilograms divided by height in meters squared); NA, not applicable; wOR, weighted odds ratio.

^a^ Calculated for a 1-unit increase in BMI.

^b^ This model was adjusted for grade, sex, BMI, residence, smoking status, current alcohol use, highest educational level of parents, economic level, and school performance.

^c^ This model was adjusted for grade, sex, BMI, residence, smoking status, current alcohol use, highest educational level of parents, economic level, and school performance.

## Discussion

### Findings and Explanation

This study analyzed trends and associated factors of sadness and suicidality during the COVID-19 pandemic compared with the prepandemic period using nationally representative survey data of over 1 million South Korean adolescents from 2005 to 2021. To our knowledge, this is the first long-term, large-scale study using a data set of over 1 million adolescents (including 106 979 adolescents during the pandemic) to examine trends and associated factors regarding sadness and suicidality. The prevalence rates of sadness and suicidality showed a steady decrease over time, with different slopes except during the COVID-19 pandemic. Although the overall prevalence of alcohol and smoking declined over the period, those who engaged in drinking and smoking behaviors showed increased sadness and suicidality during the pandemic. Younger age, female sex, urban residence, smoking, current alcohol use, and low economic status were risk factors significantly associated with sadness and suicidality during the pandemic. These results are in line with a previous study that suggested the pandemic may have an effect on vulnerable groups.^24^

### Plausible Mechanism

Our study showed that sadness and suicidality rates increased during the midpandemic period. As the COVID-19 pandemic has persisted, the nation’s financial support for the economy steadily decreased.^7,25^ Adolescents worry about infecting themselves and their families, occasionally find social quarantine measures annoying, worry about when the pandemic will end, and feel alone as a result of the prolonged closure of schools and other public places. In addition, adolescents worry about the lack of opportunities to socialize outside.^26^ Adolescents have been significantly affected psychologically by the COVID-19 pandemic; thus, it is important to assess trends over time to evaluate whether there is an association between the pandemic and sadness or suicidality in the pandemic period after 2021.

The risk factors for sadness and suicidality during the pandemic were young age, female sex, urban residence, smoking status, current alcohol use, and low economic status. Duan et al^26^ found that urban inhabitants were more anxious than those in rural regions because COVID-19 outbreaks began in very densely populated areas. In comparison with male youths, female youths had higher levels of sadness and suicidality during the COVID-19 period, possibly because they may be more vulnerable to stressful life events.^27^ In addition, adolescents with risk factors for sadness and suicidality are more prone to becoming victims of domestic violence, including child abuse or relational violence, which became increasingly common during times of lockdown and stay-at-home orders, and might also have caused an increase in alcohol consumption.^28,29^

### Comparison With Previous Studies

The COVID-19 pandemic has been shown to have a significant negative association with the mental health of adolescents in several studies,^30,31,32^ with most of the research focusing on China (n = 859 to 8079),^26,33,34,35,36,37,38,39^ Canada (n = 1054),^40^ the US (n = 1181),^41^ Spain (n = 459 to 1049),^42,43^ Brazil (n = 289),^44^ Greece (n = 67),^45^ India (n = 121),^46^ Israel (n = 351),^47^ and Bangladesh (n = 384).^48^ However, these studies may have produced low levels of evidence and conflicting results because of their small sample sizes, short follow-up periods (most were only conducted up to 2020), and inadequate study designs (nonrepresentative or nonrandom selection of participants, including convenience, purposive, and volunteer sampling).^49^ In contrast, our study used long-term, large population-based data from a nationwide investigation to examine the trends and associated factors of adolescents’ sadness and suicidality in South Korea from 2005 to 2021.

### Policy Implications

Adolescence is a critical stage of life when a person experiences a rapid range of physical and psychological changes, creating both opportunities and dangers for healthy growth.^50^ Adolescents’ investment in mental health and well-being is beneficial to the present and next generations.^50^ Understanding the trends and risk factors of adolescents’ mental health issues during the pandemic period is important to identify vulnerable adolescents and provide programs that aim to address mental health problems.^51^ Because the risk factors vary depending on individual circumstances, such as school grade and current alcohol use during the pandemic for suicidality, it is imperative to adopt approaches that optimize the limited resources available and consider factors such as substance abuse, urban residency, and vulnerable adolescents, including those of younger age, female sex, and lower economic status.

### Limitations

This study has several limitations. First, a self-report questionnaire was used to collect information about adolescent sadness and suicidality, which may underestimate the prevalence of these issues due to reporting bias (eg, recall, information, and selection biases) and the stigma effect. Second, Korean adolescents’ degree of sadness is unknown. In addition, there has been no investigation of self-injury or overdose, which further limits our understanding. Third, our data pertain to Korean adolescents only during the period when strict pandemic precautions were in place from 2020 to 2021,^52^ and sexual minority status (self-identification as lesbian, gay, bisexual, transgender, queer, or questioning) was not investigated; the findings may not be generalizable to other regions of the world. Large-scale international research is required to examine the factors associated with adolescents’ sadness and suicidality. Fourth, this study did not account for 35 402 (3.2%) cases of missing information, which may have been associated with our findings. In future studies, missing data can be imputed using multiple imputation methods. Fifth, the survey on economic status and academic performance was divided into 5 categories based on students’ experience rather than actual income. Evaluating the association between economic status, academic performance, and sadness or suicidality objectively is crucial as a predictor of the association of familial economic status. Sixth, in this study, we found that younger age, female sex, urban residence, smoking, current alcohol use, and low economic status were associated with an increased risk of sadness and suicidality. Although other government policy factors, such as quarantine, school change, and social opportunities, may also be risk factors, we were unable to confirm their association with sadness and suicidality in our study. Therefore, in addition to the risk factors presented here, other factors should also be considered. Seventh, this study needs to be continued to consider risk factors after the COVID-19 pandemic ends. Despite these limitations, this study is the first long-term, large-scale study using a data set of more than 1 million adolescents to examine trends and factors associated with sadness and suicidality.

## Conclusion

In this cross-sectional survey study of South Korean adolescents, the slope of the prevalence of sadness and suicidality increased during the COVID-19 pandemic after a decrease prior to the pandemic. This was the first long-term, large-scale, serial cross-sectional study to examine trends and factors associated with sadness and suicidality among over 1 million single-ethnicity South Korean adolescents. Younger age, female sex, urban residence, smoking status, current alcohol use, and low economic status were risk factors for sadness and suicidality during the COVID-19 pandemic. These findings indicated that various factors associated with adolescent sadness and suicidality interact intricately. Our study suggests that public health measures need to be developed for recognizing vulnerable groups with risk factors and preventing an increase in sadness and suicidality among adolescents during the COVID-19 pandemic.

## Notes

Supplement 1. **eFigure 1.** The Method of Calculating the Weight **eFigure 2.** Nationwide 17-Year Trends and Prevalence of Sadness and Suicidality Among One Million Korean Adolescents, 2005-2021 **eTable 1.** Demographic Characteristics of Participating Adolescents in the KYRBS, 2005-2021 (total n=1,109,776) **eTable 2.** Univariate Logistic Regression Analysis for Sadness **eTable 3.** Univariate Logistic Regression Analysis for Suicidality **eTable 4.** Adjusted and Logistic Regression Analysis for Sadness, 2005-2021 **eTable 5.** Adjusted and Logistic Regression Analysis for Suicidality, 2005-2021

Supplement 2.
Data Sharing Statement
