# Supplementary material for: National Trends in Sadness, Suicidality, and COVID-19 Pandemic–Related Risk Factors Among South Korean Adolescents From 2005 to 2021
Source: JAMA Netw Open. 2023 May 24;6(5):e2314838. doi: 10.1001/jamanetworkopen.2023.14838 (PMC10209749; doi:10.1001/jamanetworkopen.2023.14838)
Supplement: Supplement 1. — eFigure 1. The Method of Calculating the Weight eFigure 2. Nationwide 17-Year Trends and Prevalence of Sadness and Suicidality Among One Million Korean Adolescents, 2005-2021 eTable 1. Demographic Characteristics of Participating Adolescents in the KYRBS, 2005-2021 (total n=1,109,776) eTable 2. Univariate Logistic Regression Analysis for Sadness eTable 3. Univariate Logistic Regression Analysis for Suicidality eTable 4. Adjusted and Logistic Regression Analysis for Sadness, 2005-2021 eTable 5. Adjusted and Logistic Regression Analysis for Suicidality, 2005-2021 [file jamanetwopen-e2314838-s001.pdf]

Supplementary Online Content

Woo HG, Park S, Yon H, et al. National trends in sadness, suicidality, and COVID-19 pandemic–related risk factors among South Korean adolescents from 2005 to 2021. *JAMA Netw Open.* 2023;6(5):e2314838. doi:10.1001/jamanetworkopen.2023.14838

eFigure 1. The Method of Calculating the Weight
eFigure 2. Nationwide 17-Year Trends and Prevalence of Sadness and Suicidality Among One Million Korean Adolescents, 2005-2021
eTable 1. Demographic Characteristics of Participating Adolescents in the KYRBS, 2005-2021 (total n=1,109,776)
eTable 2. Univariate Logistic Regression Analysis for Sadness
eTable 3. Univariate Logistic Regression Analysis for Suicidality
eTable 4. Adjusted and Logistic Regression Analysis for Sadness, 2005-2021
eTable 5. Adjusted and Logistic Regression Analysis for Suicidality, 2005-2021

This supplementary material has been provided by the authors to give readers additional information about their work.

© 2023 Woo HG et al. *JAMA Network Open.*

eFigure 1. The Method of Calculating the Weight

The weight was calculated by the Korea Centers for Disease Control and Prevention and is provided as a weight variable (W) in raw data. The weight is a value obtained by multiplying the inverse of the extraction rate and the inverse of the response rate by the post-calibration rate of the weight.

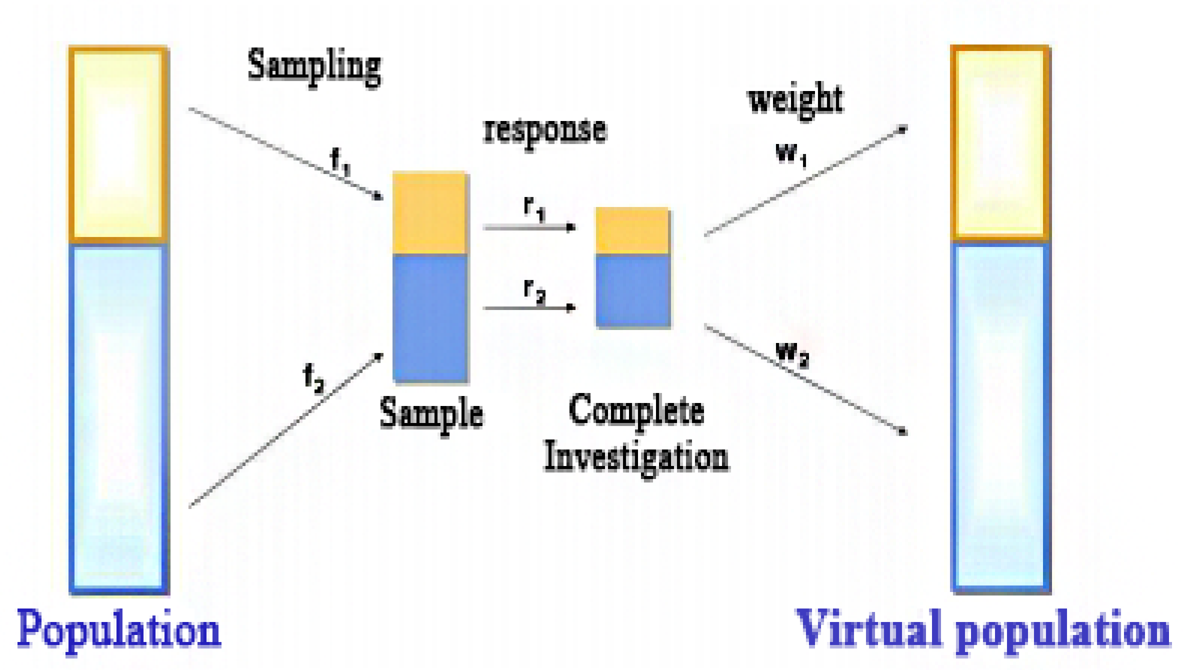

© 2023 Woo HG et al. *JAMA Network Open.*

eFigure 2. Nationwide 17-Year Trends and Prevalence of Sadness and Suicidality Among One Million Korean Adolescents, 2005-2021

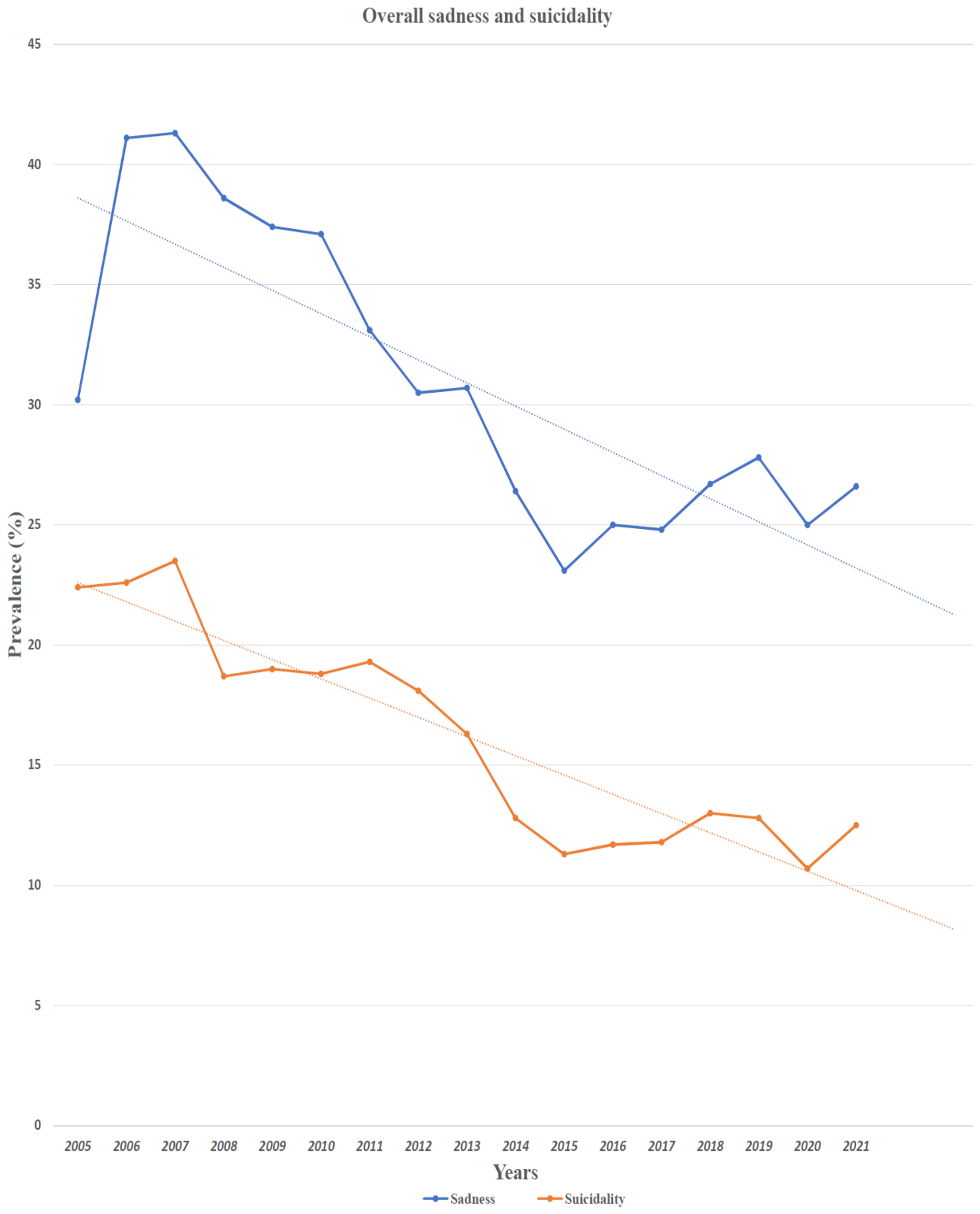

© 2023 Woo HG et al. *JAMA Network Open.*

eTable 1. Demographic Characteristics of Participating Adolescents in the KYRBS, 2005-2021 (total n=1,109,776)

Variables	Crude sample					
	Overall	2005 to 2007	2008 to 2011	2012 to 2015	2016 to 2019	COVID-19 pandemic (2020–2021)
Number, (%)	1,109,776 (100.0)	196,586 (17.7)	289,384 (26.1)	278,610 (25.1)	238,217 (21.5)	106,979 (9.6)
Age, years, mean ± SD	14.99 ± 1.74	14.93 ± 1.68	15.05 ± 1.75	14.92 ± 1.75	14.99 ± 1.76	15.09 ± 1.75
Grade, number (%)						
7th-9th grade (middle school)	573,932 (51.7)	107,463 (54.7)	148,735 (51.4)	140,502 (50.4)	119,621 (50.2)	57,611 (53.9)
10th-12th grade (high school)	535,844 (48.3)	89,123 (45.3)	140,649 (48.6)	138,108 (49.6)	118,596 (49.8)	49,368 (46.1)
Sex, number (%)						
Male	572,055 (51.5)	102,107 (51.9)	149,903 (51.8)	142,347 (51.1)	122,238 (51.3)	55,460 (51.8)
Female	537,721 (48.5)	94,479 (48.1)	139,481 (48.2)	136,263 (48.9)	115,979 (48.7)	51,519 (48.2)
BMI, kg/m^2, mean ± SD	20.81 ± 3.21	20.50 ± 2.99	20.48 ± 2.97	20.72 ± 3.09	21.22 ± 3.44	21.57 ± 3.75
Residence, number (%)						

© 2023 Woo HG et al. *JAMA Network Open.*

Rural	514,496 (46.4)	94,189 (47.9)	143,301 (49.5)	124,821 (44.8)	105,787 (44.4)	46,398 (43.4)
Urban	595,280 (53.6)	102,397 (52.1)	146,083 (50.5)	153,789 (55.2)	132,430 (55.6)	60,581 (56.6)
Smoking, number (%)	103,793 (9.4)	24,302 (12.4)	35,370 (12.2)	25,384 (9.1)	14,113 (5.9)	4,624 (4.3)
Current alcohol use, number (%)	211,679 (19.1)	55,263 (28.1)	63,036 (21.8)	46,820 (16.8)	35,338 (14.8)	11,222 (10.5)
Sadness, number (%)	347,456 (31.3)	74,785 (38.0)	105,685 (36.5)	77,385 (27.8)	61,979 (26.0)	27,622 (25.8)
Suicidality, number (%)	182,421 (16.4)	44,984 (22.9)	54,830 (18.9)	40,938 (14.7)	29,268 (12.3)	12,401 (11.6)
Highest educational level of parents, number (%)						
High school or lower	521,635 (47.0)	128,261 (65.2)	166,015 (57.4)	128,176 (46.0)	75,785 (31.8)	23,398 (21.9)
College or higher	409,612 (36.9)	47,439 (24.1)	91,016 (31.5)	113,366 (40.7)	111,109 (46.6)	46,682 (43.6)
Unknown	178,529 (16.1)	20,886 (10.6)	32,353 (11.2)	37,068 (13.3)	51,323 (21.5)	36,899 (34.5)
Economic level, number (%)						
High	87,663 (7.9)	13,312 (6.8)	17,309 (6.0)	20,768 (7.5)	24,669 (10.4)	11,605 (10.8)
Middle-high	291,759 (26.3)	58,310 (29.7)	64,395 (22.3)	70,172 (25.2)	68,497 (28.8)	30,385 (28.4)
Middle	523,241 (47.1)	87,735 (44.6)	137,681 (47.6)	133,319 (47.9)	112,330 (47.2)	52,176 (48.8)
Middle-low	163,052 (14.7)	29,192 (14.8)	52,948 (18.3)	43,167 (15.5)	27,131 (11.4)	10,614 (9.9)

© 2023 Woo HG et al. *JAMA Network Open.*

Low	44,061 (4.0)	8,037 (4.1)	17,051 (5.9)	11,184 (4.0)	5,590 (2.3)	2,199 (2.1)
School performance, number (%)						
High	135,726 (12.2)	25,962 (13.2)	32,493 (11.2)	32,151 (11.5)	31,665 (13.3)	13,455 (12.6)
Middle-high	281,457 (25.4)	56,644 (28.8)	69,136 (23.9)	68,256 (24.5)	60,999 (25.6)	26,422 (24.7)
Middle	316,853 (28.6)	58,294 (29.7)	78,687 (27.2)	77,424 (27.8)	69,677 (29.2)	32,771 (30.6)
Middle-low	259,611 (23.4)	40,334 (20.5)	73,975 (25.6)	68,261 (24.5)	53,092 (22.3)	23,949 (22.4)
Low	116,129 (10.5)	15,352 (7.8)	35,093 (12.1)	32,518 (11.7)	22,784 (9.6)	10,382 (9.7)

BMI, body mass index; CI, confidence interval; KYRBS, Korea Youth Risk Behavior Web-based Survey; SD, standard deviation.

© 2023 Woo HG et al. *JAMA Network Open.*

eTable 2. Univariate Logistic Regression Analysis for Sadness

Variables	Overall (n=1,109,776)		Pre-COVID-19 pandemic (n=1,002,797)		COVID-19 pandemic (n=106,979)		Ratio of OR (Pandemic/Pre) (95% CI)	*p*-value
	Crude OR (95% CI)	*p*-value	OR (95% CI)	*p*-value	OR (95% CI)	*p*-value		
Age	1.087 (1.085 to 1.090)	<0.001	1.091 (1.089 to 1.094)	<0.001	1.058 (1.050 to 1.066)	<0.001	0.970 (0.962 to 0.977)	<0.001
Grade								
7th-9th grade (middle school)	1.000 (reference)		1.000 (reference)		1.000 (reference)			
10th-12th grade (high school)	1.262 (1.252 to 1.272)	<0.001	1.270 (1.259 to 1.281)	<0.001	1.152 (1.121 to 1.184)	<0.001	0.907 (0.881 to 0.933)	<0.001
Sex								
Male	1.000 (reference)		1.000 (reference)		1.000 (reference)			
Female	1.635 (1.622 to 1.648)	<0.001	1.630 (1.616 to 1.644)	<0.001	1.701 (1.654 to 1.749)	<0.001	1.044 (1.014 to 1.074)	0.004
BMI	0.995	<0.001	0.998	0.006	0.992	<0.001	0.994	0.001

© 2023 Woo HG et al. *JAMA Network Open.*

	(0.994 to 0.997)		(0.997 to 0.999)		(0.988 to 0.995)		(0.990 to 0.998)	
Residence								
Rural	1.008 (1.000 to 1.017)	0.04	1.015 (1.006 to 1.023)	0.001	0.906 (0.881 to 0.931)	<0.001	0.893 (0.867 to 0.919)	<0.001
Urban	1.000 (reference)		1.000 (reference)		1.000 (reference)			
Smoking	2.061 (2.035 to 2.088)	<0.001	2.016 (1.989 to 2.042)	<0.001	2.438 (2.297 to 2.588)	<0.001	1.209 (1.137 to 1.286)	<0.001
Current alcohol use	2.003 (1.984 to 2.023)	<0.001	1.975 (1.955 to 1.995)	<0.001	2.078 (1.995 to 2.164)	<0.001	1.052 (1.009 to 1.097)	0.02
Highest educational level of parents								
High school or lower	1.000 (reference)		1.000 (reference)		1.000 (reference)			
College or higher	1.161 (1.151 to 1.171)	<0.001	1.141 (1.131 to 1.152)	<0.001	1.129 (1.090 to 1.170)	<0.001	0.989 (0.954 to 1.026)	0.57
Unknown	0.861 (0.850 to 0.872)	<0.001	0.863 (0.851 to 0.874)	<0.001	0.965 (0.935 to 0.996)	0.03	1.118 (1.080 to 1.157)	<0.001

© 2023 Woo HG et al. *JAMA Network Open.*

Economic level								
High	1.000 (reference)		1.000 (reference)		1.000 (reference)			
Middle-high	1.038 (1.021 to 1.056)	<0.001	1.033 (1.014 to 1.052)	<0.001	1.022 (0.972 to 1.075)	0.39	0.989 (0.938 to 1.044)	0.70
Middle	1.113 (1.095 to 1.131)	<0.001	1.111 (1.093 to 1.130)	<0.001	1.048 (1.000 to 1.099)	0.05	0.943 (0.906 to 0.982)	0.004
Middle-low	1.692 (1.662 to 1.722)	<0.001	1.670 (1.639 to 1.702)	<0.001	1.646 (1.552 to 1.745)	<0.001	0.986 (0.927 to 1.048)	0.65
Low	2.510 (2.451 to 2.571)	<0.001	2.466 (2.405 to 2.528)	<0.001	2.509 (2.283 to 2.758)	<0.001	1.017 (0.923 to 1.122)	0.73
School performance								
High	1.000 (reference)		1.000 (reference)		1.000 (reference)			
Middle-high	1.128 (1.112 to 1.145)	<0.001	1.132 (1.115 to 1.149)	<0.001	1.074 (1.022 to 1.129)	0.005	0.949 (0.901 to 0.999)	0.04
Middle	1.237 (1.219 to 1.255)	<0.001	1.247 (1.228 to 1.265)	<0.001	1.159 (1.105 to 1.216)	<0.001	0.929 (0.884 to 0.977)	0.004

© 2023 Woo HG et al. *JAMA Network Open.*

Middle-low	1.552 (1.529 to 1.574)	<0.001	1.556 (1.533 to 1.580)	<0.001	1.482 (1.410 to 1.557)	<0.001	0.952 (0.904 to 1.003)	0.07
Low	1.999 (1.966 to 2.033)	<0.001	1.995 (1.960 to 2.031)	<0.001	2.005 (1.894 to 2.124)	<0.001	1.005 (0.946 to 1.067)	0.87

BMI, body mass index.

The odds ratio was calculated for 1 unit increase in BMI (1kg/m^2).

Numbers in bold indicate a significant difference ($p < 0.05$).

© 2023 Woo HG et al. *JAMA Network Open.*

eTable 3. Univariate Logistic Regression Analysis for Suicidality

Variables	Overall (n=1,109,776)		Pre-COVID-19 pandemic (n=1,002,797)		COVID-19 pandemic (n=106,979)		Ratio of OR (Pandemic/Pre) (95% CI)	*p*-value
	Crude OR (95% CI)	*p*-value	OR (95% CI)	*p*-value	OR (95% CI)	*p*-value		
Age	1.000 (0.997 to 1.003)	0.97	1.001 (0.998 to 1.004)	0.46	1.003 (0.992 to 1.014)	0.59	1.002 (0.991 to 1.013)	0.73
Grade								
7th-9th grade (middle school)	1.000 (reference)		1.000 (reference)		1.000 (reference)			
10th-12th grade (high school)	0.965 (0.955 to 0.975)	<0.001	0.960 (0.950 to 0.970)	<0.001	0.981 (0.945 to 1.018)	0.31	1.022 (0.983 to 1.062)	0.27
Sex								
Male	1.000 (reference)		1.000 (reference)		1.000 (reference)			
Female	1.728 (1.711 to 1.746)	<0.001	1.717 (1.699 to 1.735)	<0.001	1.893 (1.822 to 1.967)	<0.001	1.103 (1.060 to 1.147)	<0.001
BMI	0.998	0.02	1.001	0.34	1.002	0.40	1.001	0.71

© 2023 Woo HG et al. *JAMA Network Open.*

	(0.997 to 1.000)		(0.999 to 1.002)		(0.997 to 1.007)		(0.996 to 1.006)	
Residence								
Rural	1.020 (1.010 to 1.031)	<0.001	1.024 (1.013 to 1.034)	<0.001	0.920 (0.886 to 0.956)	<0.001	0.898 (0.864 to 0.935)	<0.001
Urban	1.000 (reference)		1.000 (reference)		1.000 (reference)			
Smoking	2.056 (2.026 to 2.087)	<0.001	2.003 (1.973 to 2.033)	<0.001	2.359 (2.196 to 2.533)	<0.001	1.178 (1.096 to 1.265)	<0.001
Current alcohol use	1.917 (1.895 to 1.939)	<0.001	1.874 (1.852 to 1.897)	<0.001	2.071 (1.969 to 2.179)	<0.001	1.105 (1.050 to 1.164)	<0.001
Highest educational level of parents								
High school or lower	1.000 (reference)		1.000 (reference)		1.000 (reference)			
College or higher	1.159 (1.146 to 1.172)	<0.001	1.134 (1.122 to 1.147)	<0.001	1.093 (1.042 to 1.146)	<0.001	0.964 (0.918 to 1.012)	0.14
Unknown	0.876 (0.863 to 0.890)	<0.001	0.909 (0.893 to 0.924)	<0.001	0.882 (0.844 to 0.921)	<0.001	0.970 (0.926 to 1.017)	0.21

© 2023 Woo HG et al. *JAMA Network Open.*

Economic level								
High	1.000 (reference)		1.000 (reference)		1.000 (reference)			
Middle-high	1.023 (1.001 to 1.045)	0.04	1.001 (0.979 to 1.024)	0.90	1.119 (1.041 to 1.203)	0.002	1.118 (1.036 to 1.206)	0.004
Middle	1.064 (1.042 to 1.086)	<0.001	1.041 (1.019 to 1.063)	<0.001	1.152 (1.077 to 1.234)	<0.001	1.107 (1.031 to 1.188)	0.005
Middle-low	1.740 (1.702 to 1.780)	<0.001	1.669 (1.630 to 1.708)	<0.001	2.156 (1.992 to 2.334)	<0.001	1.292 (1.189 to 1.403)	<0.001
Low	2.742 (2.667 to 2.819)	<0.001	2.624 (2.550 to 2.701)	<0.001	3.277 (2.923 to 3.674)	<0.001	1.249 (1.110 to 1.405)	<0.001
School performance								
High	1.000 (reference)		1.000 (reference)		1.000 (reference)			
Middle-high	1.044 (1.025 to 1.063)	<0.001	1.045 (1.025 to 1.066)	<0.001	0.999 (0.933 to 1.069)	0.97	0.956 (0.891 to 1.026)	0.21
Middle	1.078 (1.058 to 1.097)	<0.001	1.087 (1.067 to 1.108)	<0.001	0.992 (0.929 to 1.060)	0.82	0.913 (0.852 to 0.977)	0.009

© 2023 Woo HG et al. *JAMA Network Open.*

Middle-low	1.381<br>(1.356 to 1.406)	<0.001	1.384<br>(1.358 to 1.410)	<0.001	1.304<br>(1.220 to 1.395)	<0.001	0.942<br>(0.879 to 1.010)	0.09
Low	1.824<br>(1.787 to 1.862)	<0.001	1.819<br>(1.781 to 1.858)	<0.001	1.824<br>(1.692 to 1.967)	<0.001	1.003<br>(0.927 to 1.084)	0.95

BMI, body mass index.

The odds ratio was calculated for 1 unit increase in BMI (1kg/m^2).

Numbers in bold indicate a significant difference ($p < 0.05$).

© 2023 Woo HG et al. *JAMA Network Open.*

8 **eTable 4.** Adjusted and Logistic Regression Analysis for Sadness, 2005-2021

Variables	Overall (n=1,109,776)	
	OR (95% CI)	*p*-value
Survey cycle		
2005 to 2007	1.747 (1.703 to 1.793)	<0.001
2008 to 2011	1.644 (1.606 to 1.684)	<0.001
2012 to 2015	1.106 (1.080 to 1.133)	<0.001
2016 to 2019	1.017 (0.992 to 1.042)	0.18
2020 to 2021 (Pandemic period)	1.000 (reference)	
Grade		
7th-9th grade (middle school)	1.000 (reference)	
10th-12th grade (high school)	1.262 (1.252 to 1.272)	<0.001
Sex		
Male	1.000 (reference)	
Female	1.757 (1.742 to 1.772)	<0.001
BMI	0.997 (0.996 to 0.999)	<0.001
Residence		
Rural	1.000 (reference)	
Urban	0.973 (0.964 to 0.890)	<0.001
Smoking	1.556 (1.533 to 1.580)	<0.001
Current alcohol use	1.687 (1.668 to 1.706)	<0.001
Highest educational level of parents		

© 2023 Woo HG et al. *JAMA Network Open.*

High school or lower	1.000 (reference)	
College or higher	0.969 (0.960 to 0.978)	<0.001
Unknown	0.792 (0.781 to 0.802)	<0.001
Economic level		
High	1.000 (reference)	
Middle-high	0.957 (0.940 to 0.974)	<0.001
Middle	0.948 (0.932 to 0.964)	<0.001
Middle-low	1.343 (1.318 to 1.369)	<0.001
Low	1.857 (1.810 to 1.904)	<0.001
School performance		
High	1.000 (reference)	
Middle-high	1.087 (1.070 to 1.103)	<0.001
Middle	1.171 (1.154 to 1.189)	<0.001
Middle-low	1.369 (1.347 to 1.390)	<0.001
Low	1.601 (1.572 to 1.630)	<0.001

BMI, body mass index.

The odds ratio was calculated for 1 unit increase in BMI ($1kg/m^2$).

This model was adjusted for survey cycle, age, grade, sex, BMI, smoking status, current alcohol use, parents' highest educational level, economic level, and school performance.

Numbers in bold indicate a significant difference ($p < 0.05$).

© 2023 Woo HG et al. *JAMA Network Open.*

16 **eTable 5.** Adjusted and Logistic Regression Analysis for Suicidality, 2005-2021

Variables	Overall (n=1,109,776)	
	Crude OR (95% CI)	*p*-value
Survey cycle		
2005 to 2007	2.273 (2.202 to 2.346)	<0.001
2008 to 2011	1.780 (1.727 to 1.836)	<0.001
2012 to 2015	1.311 (1.272 to 1.352)	<0.001
2016 to 2019	1.069 (1.036 to 1.103)	<0.001
2020 to 2021 (Pandemic period)	1.000 (reference)	
Grade		
7th-9th grade (middle school)	1.000 (reference)	
10th-12th grade (high school)	0.751 (0.743 to 0.760)	<0.001
Sex		
Male	1.000 (reference)	
Female	1.904 (1.884 to 1.925)	<0.001
BMI	1.011 (1.010 to 1.013)	<0.001
Residence		
Rural	1.045 (1.035 to 1.056)	<0.001
Urban	1.000 (reference)	
Smoking	1.689 (1.660 to 1.718)	<0.001
Current alcohol use	1.689 (1.686 to 1.732)	<0.001
Highest educational level of parents		

© 2023 Woo HG et al. *JAMA Network Open.*

High school or lower	1.000 (reference)	
College or higher	0.978 (0.967 to 0.989)	<0.001
Unknown	0.780 (0.767 to 0.793)	<0.001
Economic level		
High	1.000 (reference)	
Middle-high	0.981 (0.960 to 1.003)	0.10
Middle	0.982 (0.961 to 1.003)	0.98
Middle-low	1.535 (1.500 to 1.572)	<0.001
Low	2.275 (2.209 to 2.343)	<0.001
School performance		
High	1.000 (reference)	
Middle-high	1.008 (0.989 to 1.028)	0.40
Middle	1.028 (1.009 to 1.048)	0.004
Middle-low	1.192 (1.170 to 1.216)	<0.001
Low	1.383 (1.353 to 1.414)	<0.001

BMI, body mass index.

The odds ratio was calculated for 1 unit increase in BMI (1kg/m^2).

This model was adjusted for survey cycle, age, grade, sex, BMI, smoking status, current alcohol use, parents' highest educational level, economic level, and school performance.

Numbers in bold indicate a significant difference ($p < 0.05$).

© 2023 Woo HG et al. *JAMA Network Open.*
