# Supplementary material for: National Trends in Sadness, Suicidality, and COVID-19 Pandemic–Related Risk Factors Among South Korean Adolescents From 2005 to 2021
Source: JAMA Netw Open. 2023 May 24;6(5):e2314838. doi: 10.1001/jamanetworkopen.2023.14838 (PMC10209749; doi:10.1001/jamanetworkopen.2023.14838)
Supplement: Supplement 2. — Data Sharing Statement [file jamanetwopen-e2314838-s002.pdf]

Data Sharing Statement

Woo. National Trends in Sadness, Suicidality, and COVID-19 Pandemic–Related Risk Factors Among South Korean Adolescents From 2005 to 2021. *JAMA Netw Open*. Published May 24, 2023. doi:10.1001/jamanetworkopen.2023.14838

Data
Data available: No

Additional Information
Explanation for why data not available: Data are available on reasonable request. Study protocol, statistical code: available from DKYon (email: yonkkang@gmail.com). Data set: available from the Korea Disease Control and Prevention Agency through a data use agreement.
